# Prognostic Value of Combined Neutrophil-to-Lymphocyte Ratio and Prognostic Nutritional Index in Advanced Ovarian Cancer Treated With Neoadjuvant Chemotherapy

**DOI:** 10.7759/cureus.108250

**Published:** 2026-05-04

**Authors:** Hiroaki Ishida, Hiroki Egashira, Naruhito Asano, Hajime Yudate, Akiko Takashima, Hideaki Shimada

**Affiliations:** 1 Department of Obstetrics and Gynecology, Toho University Medical Center Sakura Hospital, Sakura, JPN; 2 Health Management Center, Japan Community Health Care Organization (JCHO) Funabashi Central Hospital, Funabashi, JPN

**Keywords:** epithelial ovarian cancer, interval debulking surgery, neoadjuvant therapy (nac), neutrophil to lymphocyte ratio (nlr), prognostic nutritional index (pni)

## Abstract

Background

Inflammation- and nutrition-related biomarkers such as the neutrophil-to-lymphocyte ratio (NLR) and prognostic nutritional index (PNI) have been reported as prognostic factors for ovarian cancer. However, their clinical significance in patients treated with neoadjuvant chemotherapy (NAC) has not been fully elucidated.

Methods

We retrospectively analyzed 25 patients with advanced ovarian cancer accompanied by peritoneal dissemination who were deemed unsuitable for complete primary debulking surgery and subsequently underwent interval debulking surgery after NAC. They were classified into the complete (R0) and incomplete (R1) resection groups based on the postoperative residual tumor status. Associations of NLR, PNI, and their combination with progression-free survival (PFS) and overall survival (OS) were evaluated.

Results

Patients who achieved complete resection (R0) had significantly longer PFS and OS than those who achieved incomplete resection (R1) (P < 0.01). Neither NLR (<5 vs. ≥5) nor PNI (>35 vs. ≤35) alone was significantly associated with PFS or OS. In contrast, patients with a high NLR (≥5) combined with a low PNI (≤35) had significantly poorer PFS (P = 0.05) and OS (P = 0.03) than the remaining patients.

Conclusions

The combination of elevated NLR and decreased PNI may serve as a useful prognostic indicator for patients with advanced ovarian cancer treated with NAC. An integrated evaluation of inflammatory and nutritional statuses may contribute to improved risk stratification and clinical decision-making in this treatment setting.

## Introduction

Advanced ovarian cancer is frequently diagnosed at an advanced stage with extensive peritoneal dissemination and remains one of the most lethal gynecological malignancies worldwide [1,2]. Despite advances in surgical techniques and systemic therapies, the long-term prognosis of patients with International Federation of Gynecology and Obstetrics (FIGO) stages III-IV disease remains unsatisfactory [2].

Maximal cytoreductive surgery is the cornerstone of initial treatment, and the extent of postoperative residual disease is recognized as the most useful prognostic factor [3]. However, a treatment strategy consisting of neoadjuvant chemotherapy (NAC) followed by interval debulking surgery (IDS) has been established as the standard approach for patients deemed ineligible for complete primary debulking surgery at initial presentation [4,5]. While this strategy improves surgical feasibility and perioperative safety, inter-individual variability in the treatment response and survival outcomes among patients receiving NAC is substantial.

Several prognostic factors for advanced ovarian cancer have been reported, including the extent of cytoreduction [3], Kinetic ELIMination (KELIM) of CA125 score based on CA125 dynamics [6], and tumor biological factors such as BRCA mutations and homologous recombination deficiency [7]. However, many of them are evaluated after surgery or during chemotherapy, limiting their applicability to pretreatment risk stratification and individualized treatment decision-making.

Recently, systemic inflammatory response and nutritional status have been recognized to play crucial roles in cancer progression and patient prognosis [8]. The neutrophil-to-lymphocyte ratio (NLR) is a representative biomarker of systemic inflammation [9], whereas the prognostic nutritional index (PNI), calculated from serum albumin concentrations and peripheral lymphocyte counts, reflects both nutritional status and immune function [8]. An elevated NLR and decreased PNI have been associated with poor survival outcomes for various malignancies, including ovarian cancer [10-12]. Both NLR and PNI can be readily calculated from the results of routine blood tests performed in daily clinical practice, allowing for prognostic assessment prior to treatment initiation [8]. These characteristics underscore their potential utility as clinically relevant biomarkers for the management of advanced ovarian cancer.

However, most previous studies have included heterogeneous populations comprising patients undergoing primary debulking surgery and those treated with NAC followed by IDS [10,11]. Studies specifically focusing on patients undergoing IDS after NAC remain scarce. Consequently, the prognostic significance of NLR and PNI in this specific clinical setting has not been sufficiently elucidated, and the superiority of the combination of inflammatory and nutritional markers over either alone in providing prognostic stratification has not been established.

Therefore, the present study aimed to evaluate the prognostic significance of the pretreatment NLR and PNI in patients with advanced ovarian cancer who were deemed ineligible for complete primary debulking surgery and subsequently underwent interval debulking surgery following neoadjuvant chemotherapy.

## Materials and methods

Study design and patients

This retrospective, single-center study was conducted with approval from the Ethics Committee of Toho University School of Medicine (approval number: T2025-167, dated March 12, 2026). Due to the retrospective design, the requirement for written informed consent was waived, and an opt-out consent procedure was implemented in accordance with institutional and national ethical guidelines.

We retrospectively analyzed 25 patients with advanced ovarian cancer accompanied by peritoneal dissemination (FIGO stage IIIC, IVA, or IVB) who were treated at our institution between January 2021 and December 2025. All patients were deemed ineligible for complete primary debulking surgery and subsequently underwent interval debulking surgery following three to four cycles of NAC. Eligibility was determined based on radiologic findings or intraoperative assessment using staging laparoscopy, which confirmed peritoneal dissemination and infeasibility of complete resection at the initial surgery. Clinical data were retrospectively collected from medical records.

Patients were excluded if they had (i) insufficient clinical or follow-up data, (ii) a history of other active malignancies, (iii) received prior treatment before referral to our institution, or (iv) severe comorbidities that could significantly affect survival outcomes.

Treatment and surgical outcomes

All patients received platinum-based NAC, and interval debulking surgery was performed on its completion. Surgical outcomes were classified as complete resection (R0), defined as no macroscopic residual tumor, or incomplete resection (R1), defined as the presence of a residual tumor.

Laboratory assessments

Peripheral blood samples collected before initiation of NAC were used for analysis. NLR and PNI were calculated as follows:

$\mathrm{NLR} = \frac{N}{L}$

$\mathrm{PNI} = 10 \times A + 0.005 \times L$

In the above equations, N is the absolute neutrophil count (cells/μL), L is the absolute lymphocyte count (cells/μL), and A is the serum albumin level (g/dL).

Based on previous reports [13-15] and clinical relevance, the following cutoff values were applied: NLR: < 5 vs. ≥ 5 and PNI: > 35 vs. ≤ 35.

Survival analysis

The initial treatment consisted of NAC (three to four cycles of taxane plus carboplatin), interval debulking surgery, and adjuvant chemotherapy (two to three cycles of taxane plus carboplatin). Progression-free survival (PFS) was defined as the interval from the completion of initial treatment to disease progression, and overall survival (OS) was defined as the interval from the initiation of initial treatment to death from any cause.

Statistical analysis

Survival curves were generated using the Kaplan-Meier method and compared between the groups using the log-rank test. Statistical significance was set at p < 0.05. All statistical analyses were performed using JMP Student’s Edition version 18 (SAS Institute Inc., Cary, NC, USA).

## Results

Patient characteristics

The baseline characteristics of the 25 patients who received NAC are summarized in Table 1.

**Table 1:** Patient characteristics. FIGO, International Federation of Gynecology and Obstetrics; HRD, homologous recombination deficiency; NLR, neutrophil-to lymphocyte ratio; PNI, prognostic nutritional index; PFS, progression-free survival; OS, overall survival.

Variable	Value
Number of patients	25
Age, median (range), years	66 (48–79)
Histologic type serous	23
Histologic type non-serous	2
Homologous recombination deficiency status positive	10
FIGO stage IIIC	18
FIGO stage IVA	2
FIGO stage IVB	5
Neutrophil-to-lymphocyte ratio, median (range)	4.7 (1.8–9.8)
Prognostic nutritional index, median (range)	38 (25–54)
Progression-free survival, median (range), months	8.3 (3.0–35.5)
Overall survival, median (range), months	14.0 (4.0–55.0)

Patients were further classified into four groups based on the combination of NLR and PNI values (Table 2): Group A: NLR < 5 and PNI > 35; Group B: NLR ≥ 5 and PNI > 35; Group C: NLR < 5 and PNI ≤ 35; and Group D: NLR ≥ 5 and PNI ≤ 35.

**Table 2:** Progression-free and overall survival stratified by the combined NLR and PNI categories. NLR, neutrophil-to-lymphocyte ratio; PNI, prognostic nutritional index.

Group	N	R0	Progression-free survival, median (range), months	Overall survival, median (range), months
A: NLR < 5 and PNI > 35	8	6	6.0 (3.4–16.9)	7.0 (4.0–18.4)
B: NLR ≥ 5 and PNI > 35	3	3	26.5 (18.0–29.2)	29.2 (14.0–55.0)
C: NLR < 5 and PNI ≤ 35	5	3	8.3 (8.3–35.5)	33.3 (20.0–35.5)
D: NLR ≥ 5 and PNI ≤ 35	9	5	6.0 (3.0–13.1)	10.9 (5.0–29.9)

The proportion of patients achieving R0 resection was higher in Groups A, B, and C (NLR < 5 or PNI > 35 group) (75%) than in Group D (NLR ≥ 5 / PNI ≤ 35 group) (56%); however, this difference was not statistically significant (Fisher’s exact test: p = 0.64) (Table 3).

**Table 3:** Comparison of surgical outcomes (R0 and R1 resection rates) between groups defined by NLR and PNI. NLR, neutrophil-to-lymphocyte ratio; PNI, prognostic nutritional index.

Variable	Groups A, B, and C: NLR < 5 or PNI > 35 (n=16)	Group D: NLR ≥ 5 and PNI ≤ 35 (n=9)	p-value
R0 resection, n (%)	12 (75%)	5 (56%)	0.64
R1 resection, n (%)	4 (25%)	4 (44%)	-

Surgical outcomes and survival

**Figure 1:**
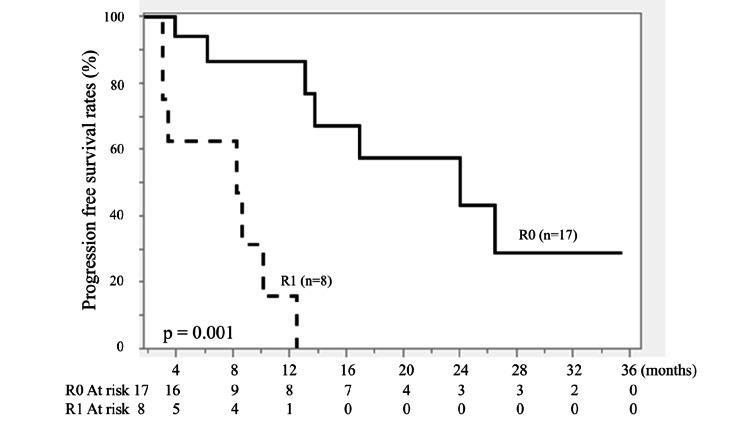
Kaplan-Meier curves of progression-free survival (PFS) for complete resection (R0, n = 17) and incomplete resection (R1, n = 8). Solid line: R0; dashed line: R1. A significant difference was observed (P = 0.001). Survival curves were generated using the Kaplan-Meier method and compared using the log-rank test.

**Figure 2:**
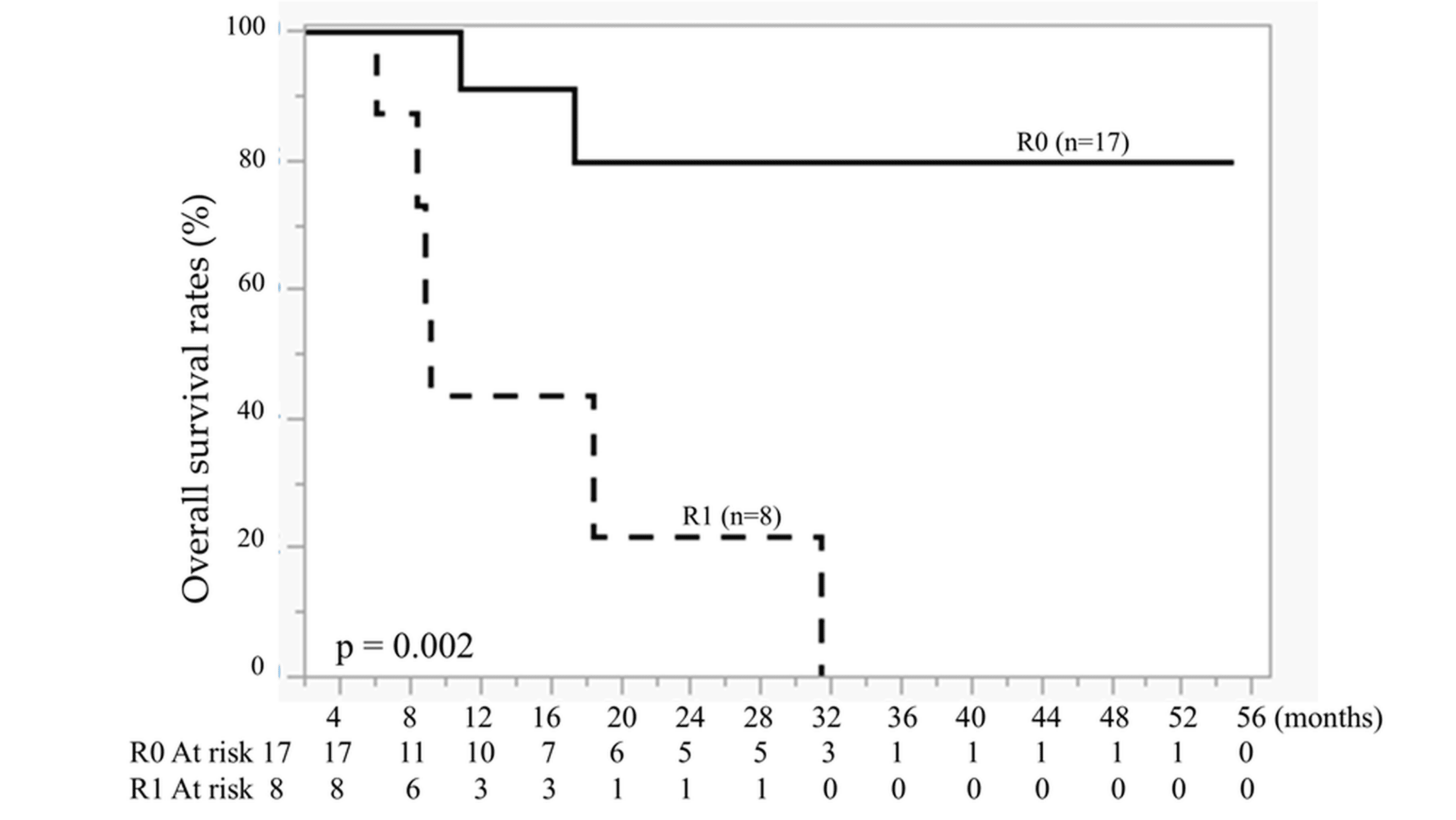
Kaplan-Meier curves of overall survival (OS) for complete resection (R0, n = 17) and incomplete resection (R1, n = 8). Solid line: R0; dashed line: R1. A significant difference was observed (P = 0.002). Survival curves were generated using the Kaplan-Meier method and compared using the log-rank test.

Complete resection (R0) was achieved in 17 patients, whereas incomplete resection (R1) was observed in eight patients. Patients in the R0 group had significantly longer PFS and OS than those in the R1 group (P < 0.01; Figures 1, 2).

Prognostic impact of NLR and PNI as individual biomarkers

**Figure 3:**
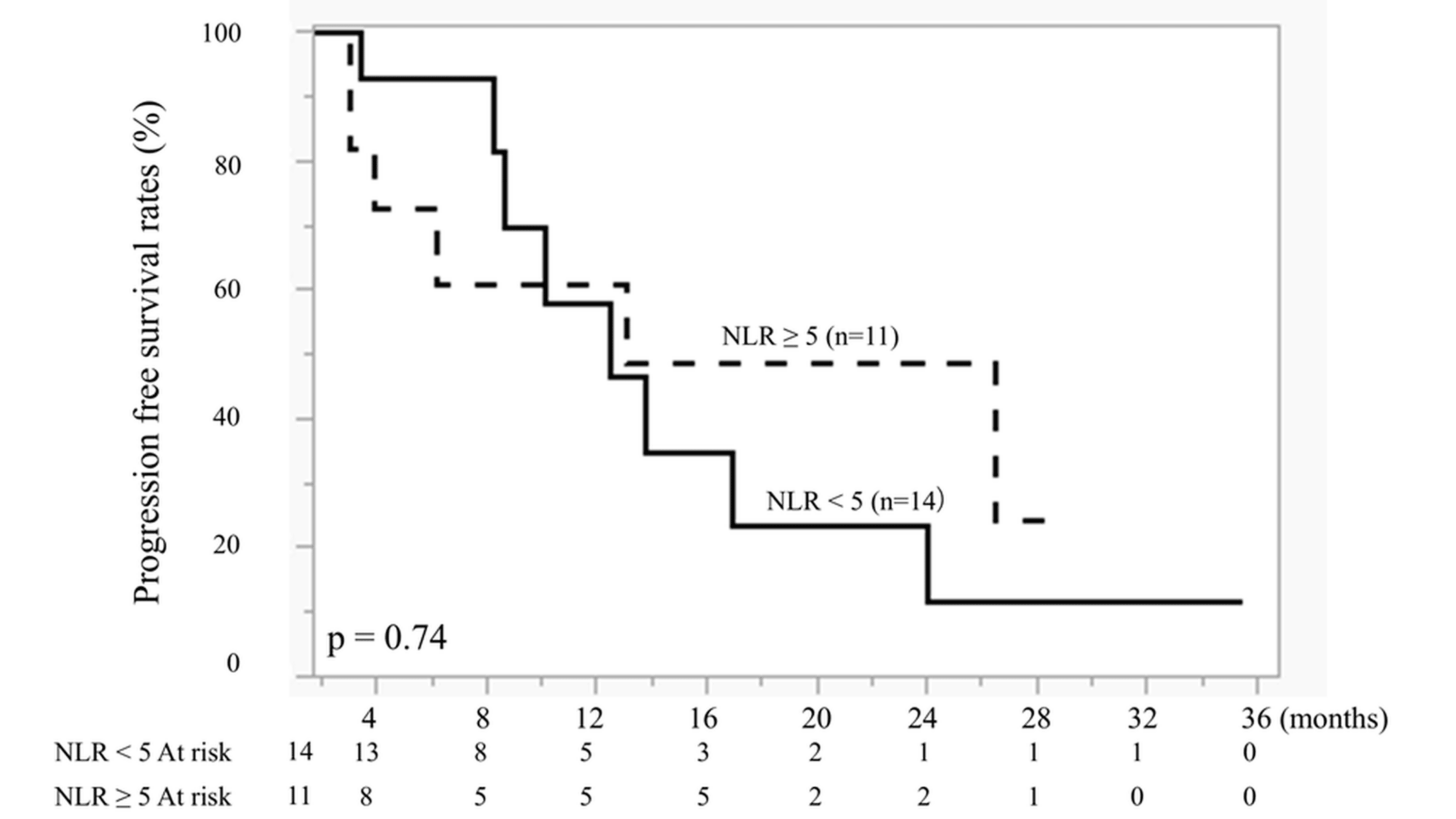
Kaplan-Meier curves of progression-free survival (PFS) according to neutrophil-to-lymphocyte ratio (NLR). Patients were stratified into NLR < 5 (n = 14) and NLR ≥ 5 (n = 11). The solid line represents NLR < 5, and the dashed line represents NLR ≥ 5. No significant difference was observed between the two groups (p = 0.74). Survival curves were generated using the Kaplan-Meier method and compared using the log-rank test.

**Figure 4:**
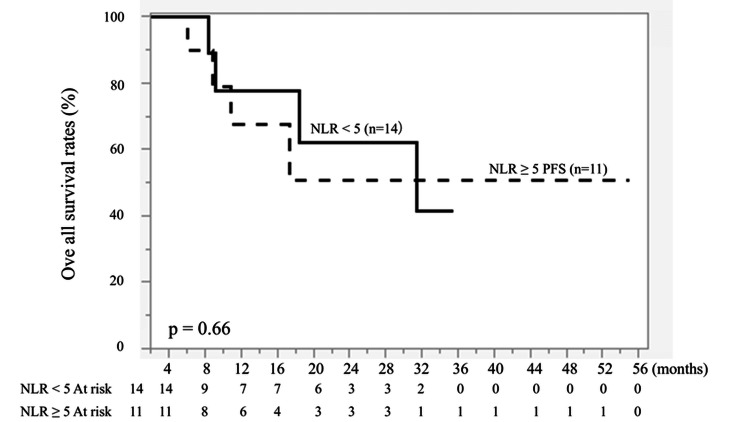
Kaplan-Meier curves of overall survival (OS) according to neutrophil-to-lymphocyte ratio (NLR). Patients were stratified into NLR < 5 (n = 14) and NLR ≥ 5 (n = 11). The solid line represents NLR < 5, and the dashed line represents NLR ≥ 5. No significant difference was observed between the two groups (P = 0.66). Survival curves were generated using the Kaplan-Meier method and compared using the log-rank test.

NLR (<5 vs. ≥5) showed no significant association with PFS (P = 0.74) or OS (P = 0.66) as an individual prognostic factor (Figures 3, 4).

**Figure 5:**
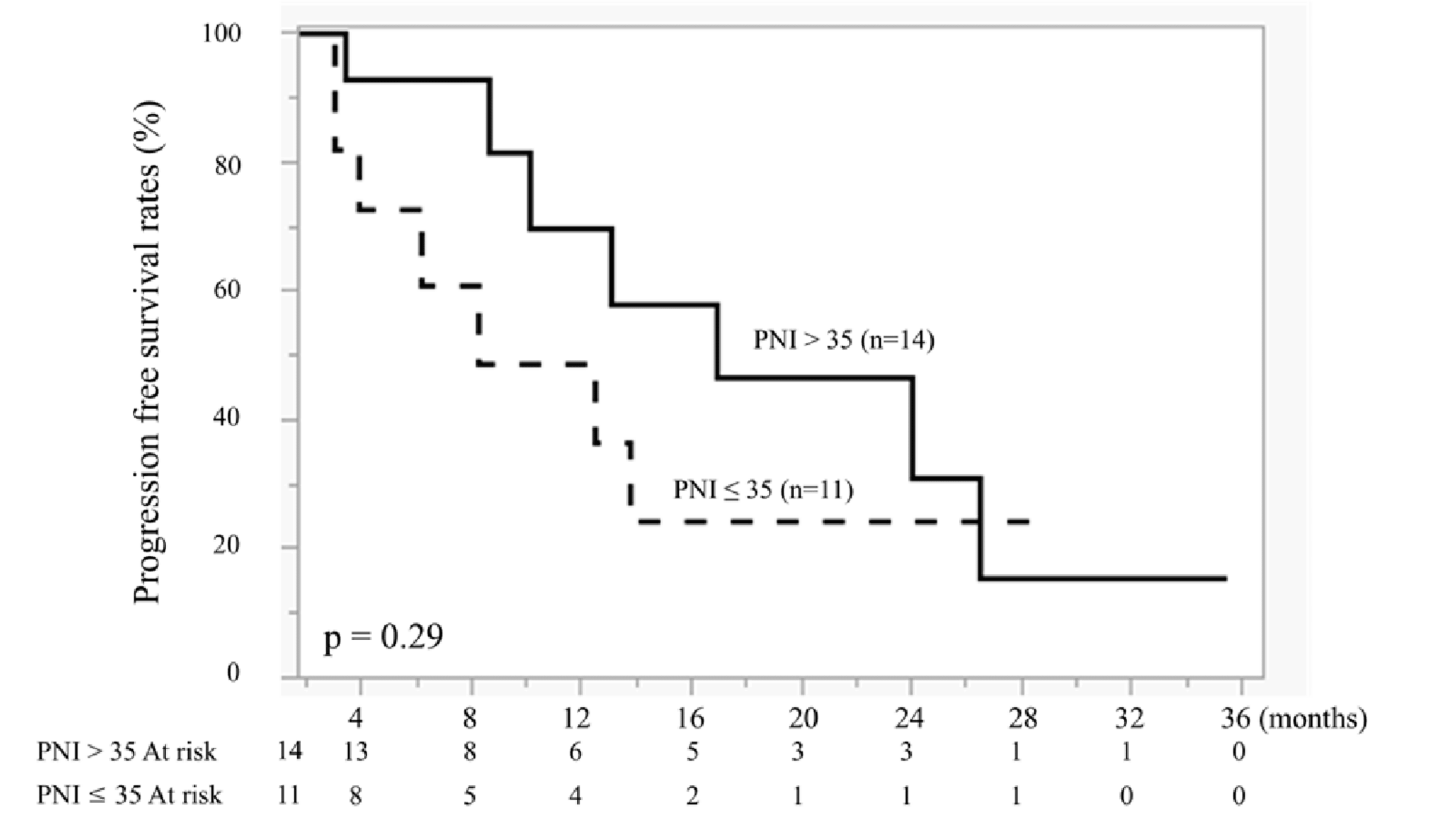
Kaplan-Meier curves of progression-free survival (PFS) according to prognostic nutritional index (PNI). Patients were stratified into PNI > 35 (n = 14) and PNI ≤ 35 (n = 11). The solid line represents PNI > 35, and the dashed line represents PNI ≤ 35. No significant difference was observed between the two groups (P = 0.29). Survival curves were generated using the Kaplan–Meier method and compared using the log-rank test.

**Figure 6:**
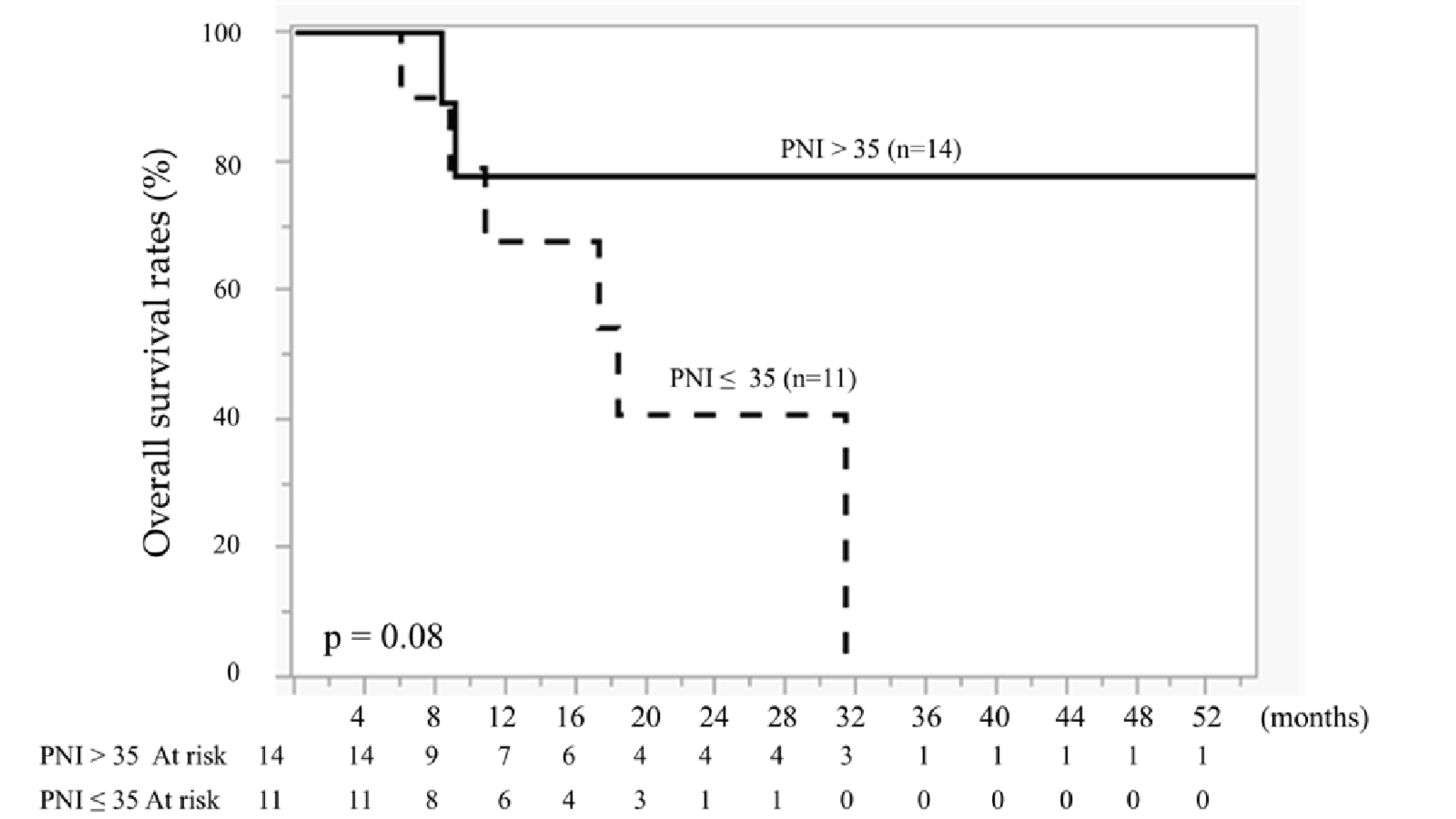
Kaplan-Meier curves of overall survival (OS) according to prognostic nutritional index (PNI). Patients were stratified into PNI > 35 (n = 14) and PNI ≤ 35 (n = 11). The solid line represents PNI > 35, and the dashed line represents PNI ≤ 35. No significant difference was observed between the two groups (P = 0.08). Survival curves were generated using the Kaplan-Meier method and compared using the log-rank test.

Similarly, PNI alone (>35 vs. ≤35) was not significantly associated with PFS (P = 0.29) or OS (P = 0.08) (Figures 5, 6).

Combined analysis of NLR and PNI

Patients were subsequently classified into two groups: Group 1: NLR < 5 or PNI > 35 (n = 16); and Group 2: NLR ≥ 5 and PNI ≤ 35 (n = 9).

**Figure 7:**
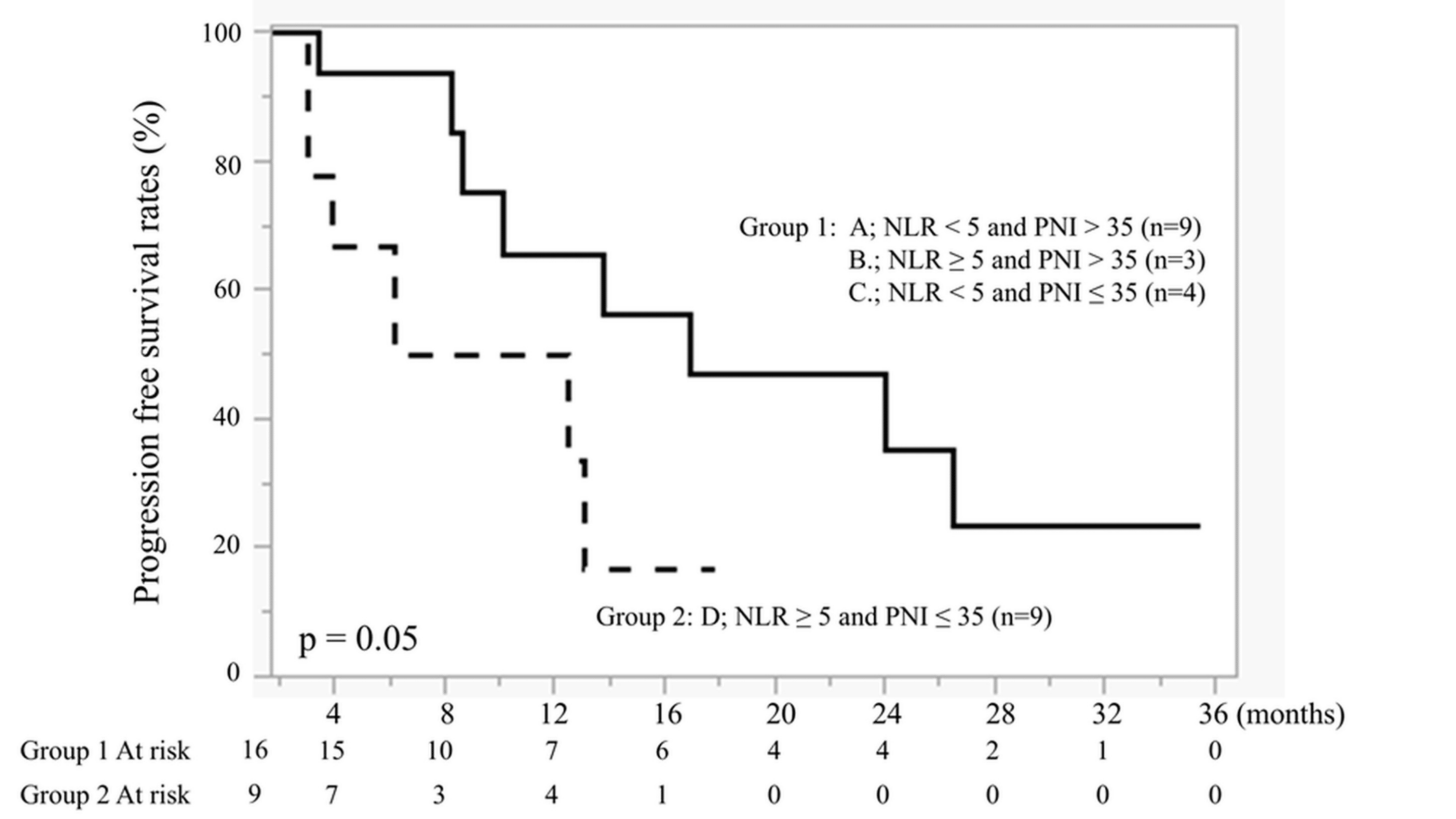
Kaplan-Meier curves of progression-free survival (PFS) based on combined NLR and PNI status. Patients were stratified into NLR < 5 or PNI > 35 (n = 16) and NLR ≥ 5 and PNI ≤ 35 (n = 9). The solid line represents NLR < 5 or PNI > 35, and the dashed line represents NLR ≥ 5 and PNI ≤ 35. A borderline significant difference was observed between the two groups (P = 0.05). Survival curves were generated using the Kaplan-Meier method and compared using the log-rank test.

**Figure 8:**
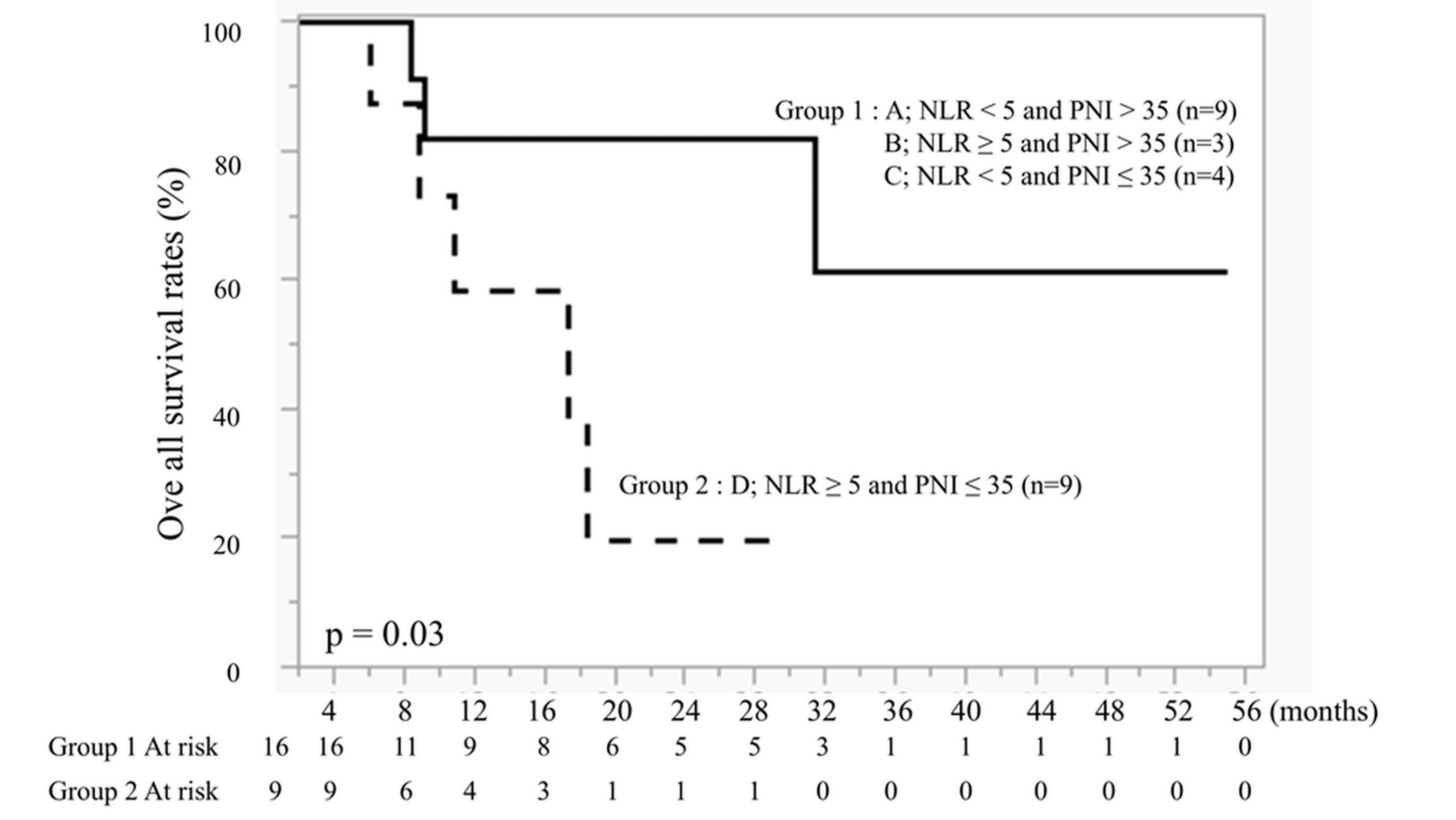
Kaplan-Meier curves of overall survival (OS) based on combined NLR and PNI status. Patients were stratified into NLR < 5 or PNI > 35 (n = 16) and NLR ≥ 5 and PNI ≤ 35 (n = 9). The solid line represents NLR < 5 or PNI > 35, and the dashed line represents NLR ≥ 5 and PNI ≤ 35. A statistically significant difference was observed between the two groups (P = 0.03). Survival curves were generated using the Kaplan-Meier method and compared using the log-rank test.

Patients in Group 2 had significantly shorter PFS (P = 0.05) and OS (P = 0.03) than those in Group 1 (Figures 7, 8).

## Discussion

In this study, we investigated the prognostic significance of pretreatment NLR and PNI in patients with advanced ovarian cancer who were deemed ineligible for complete primary debulking surgery and subsequently underwent IDS following NAC. Our results demonstrated that patients with a high NLR (≥5) and low PNI (≤35) had significantly poorer survival outcomes.

The unexpectedly short median overall survival observed in the NLR < 5 and PNI > 35 group may be attributable to the small sample size and the resulting statistical instability. In addition, survival outcomes in this cohort may have been influenced by other strong prognostic factors, such as the extent of residual disease (R0 vs R1), tumor burden, and treatment response.

Most previous studies evaluating NLR and PNI have primarily focused on patients undergoing primary debulking surgery, and evidence on populations treated with NAC followed by IDS remains limited. In contrast, the present study targeted patients with advanced ovarian cancer who were considered ineligible for resection at initial presentation and determined whether pretreatment blood-based biomarkers alone could enable risk stratification in this clinically challenging cohort. Furthermore, the novelty of this study lies in the integrated assessment of host-related factors, such as systemic inflammation (NLR) and nutritional/immune status (PNI), which are difficult to capture using a single biomarker. This allowed the identification of a composite high-risk group characterized by concomitant high NLR and low PNI.

In this study, we prioritized the use of cut-off values that could be reproducibly applied to routine clinical practice before treatment initiation. Therefore, we adopted commonly used thresholds from previous studies, setting the cutoff values at 5 for the NLR and 35 for the PNI.

The reported cutoff values for defining a high NLR for gynecologic malignancies vary widely across studies, ranging from 0.89 to 5.03 [13]. In our cohort, the median NLR was 4.7, and we selected an NLR cutoff value of 5 based on the distribution and previously published data.

For PNI, several studies have used cutoff values in the 40s [14]. However, a PNI of 35 is clinically recognized as a threshold for severe malnutrition [15]. Given that the median PNI for our cohort was 38, we adopted 35 as the cutoff value to identify patients with clinically significant nutritional impairment.

These cutoff values have been used in previous ovarian cancer studies and were intentionally selected to prioritize external validity. Excessive reliance on data-driven optimization methods, such as receiver operating characteristic curve analysis for a small cohort, carries a high risk of overfitting. Given the limited sample size of this study, identification of optimal cutoff values should be addressed in future large-scale, multicenter investigations.

The association between elevated NLR or decreased PNI and poor prognosis has been consistently reported across multiple cancer types, including ovarian cancer. However, neither NLR nor PNI alone showed a significant association with PFS or overall survival OS in this study.

This finding suggests that heterogeneity in tumor burden, systemic inflammation, and nutritional status is substantial in populations treated with NAC. Moreover, the strong prognostic impact of complete cytoreduction (R0), which remains the most powerful determinant of survival, may obscure the prognostic value of individual inflammatory and nutritional markers. Consequently, a single biomarker may be insufficient for adequate prognostic stratification in this treatment setting.

PNI reflects both nutritional status and immune competence. A low PNI is associated with malnutrition, chronic inflammation, and lymphopenia, which may impair treatment tolerance and antitumor immune responses, ultimately leading to poor survival outcomes. These mechanisms are largely consistent with those proposed for other types of malignancies.

However, ovarian cancer is strongly dependent on platinum-based systemic therapy and the extent of tumor cytoreduction. Favorable treatment responses may result in meaningful survival benefits even in patients with compromised host conditions, but such compensatory potential is not uniform. Reduced treatment intensity and early recurrence are more likely in patients with overlapping adverse host factors, such as severe malnutrition and immune dysfunction.

The NLR reflects the balance between systemic inflammation and immune surveillance. A high NLR indicates a state in which tumor-promoting inflammation is enhanced, while antitumor immune activity is suppressed, a mechanism commonly associated with poor prognosis across various malignancies. In the present study, NLR alone did not yield significant prognostic stratification, likely because of the substantial influence of treatment response, tumor burden dynamics, and surgical completeness (R0) in patients treated with NAC.

Importantly, patients with high NLR and low PNI had significantly poorer PFS and OS. This suggests that concomitant tumor-promoting inflammation, malnutrition, and immune dysfunction may synergistically reduce chemotherapy sensitivity and accelerate tumor progression [8-11]. The combined evaluation of inflammatory and nutritional markers enables a more comprehensive assessment of host status than either marker alone, which is a key finding of this study.

In addition to their prognostic value, NLR and PNI may also reflect a patient’s susceptibility to chemotherapy-related toxicity. Patients with elevated NLR and reduced PNI, indicative of systemic inflammation and malnutrition, may be more prone to hematologic toxicities such as neutropenia during neoadjuvant chemotherapy. These adverse events can lead to dose delays or reductions, potentially compromising treatment intensity. Consequently, such factors may negatively affect tumor response and the likelihood of achieving optimal cytoreduction at the time of interval debulking surgery. This potential “toxicity-to-outcome” pathway may partially explain the observed association between host-related biomarkers and survival outcomes.

From a clinical perspective, the combined assessment of NLR and PNI before NAC initiation may facilitate the identification of patients at high risk of poor treatment response or early recurrence. This information could support cautious treatment planning, optimization of treatment intensity, and consideration of multimodal therapeutic strategies. Additionally, these biomarkers are minimally invasive, cost-effective, and suitable for repeated measurements, making them highly advantageous for routine clinical practice [8].

This study has limitations. First, it was a retrospective, single-center study with a small sample size of 25 patients. Second, further validation of the optimal thresholds is warranted, although the cutoff values for NLR and PNI were selected based on previous literature and clinical relevance [13-15]. Third, this study did not incorporate molecular or biological prognostic factors such as BRCA mutation status, homologous recombination deficiency, or the Kinetic ELIMination (KELIM) of CA125 score [6,7]. Future studies integrating these factors into multivariate prognostic models are required.

In conclusion, this study suggests that the combined assessment of the NLR and PNI may serve as a useful prognostic indicator for patients with advanced ovarian cancer treated with NAC. Simple blood-based biomarkers that integrate inflammatory and nutritional statuses may contribute to pretreatment risk stratification and the development of individualized treatment strategies. Further validation in prospective multicenter studies is warranted.

## Conclusions

In this study, complete cytoreductive surgery (R0) was significantly associated with improved PFS and OS in patients with advanced ovarian cancer treated with neoadjuvant chemotherapy. While neither the NLR nor the PNI alone showed significant prognostic value, their combination identified a subgroup of patients with markedly poorer survival outcomes.

The combination of elevated NLR and decreased PNI may therefore serve as a clinically useful prognostic indicator in this specific treatment setting. This simple and readily available approach, based on routine blood tests, may facilitate pretreatment risk stratification and support individualized treatment decision-making. Further validation in larger, prospective multicenter studies is warranted to confirm these findings and to establish their clinical applicability.
